# Extensive systemic metastases from primary central nervous system haemangiopericytoma

**DOI:** 10.1259/bjrcr.20190081

**Published:** 2020-09-29

**Authors:** Chi Long Ho, Jeremy JH Lam, Robert Chun Chen

**Affiliations:** 1 Department of Radiology, Sengkang General Hospital, Singapore, Singapore; 2 Lee Kong Chian School of Medicine, Nanyang Technological University, Singapore, Singapore; 3 Duke-NUS (National University of Singapore) Graduate Medical School, Singapore, Singapore; 4 Department of Radiology, Changi General Hospital, Singapore, Singapore; 5 Department of Diagnostic Radiology, Singapore General Hospital, Singapore, Singapore

## Abstract

Primary intracranial tumours rarely metastasise outside of the central nervous system (CNS). This report describes a rare case of recurrent meningeal haemangiopericytoma with extensive systemic metastases, which eventually resulted in a fatal outcome. We discuss some prevailing theories as to the rarity of extracranial metastases from primary CNS haemangiopericytoma, and elucidate the epidemiology, imaging features, differential diagnosis, treatment, and prognosis of this unusual but surprisingly aggressive meningeal tumour. Besides aggressive treatment for local tumour control, patients with primary CNS haemangiopericytoma require long-term post-treatment surveillance to detect systemic metastases.

## Introduction

The classic teaching is that the metastatic spread between the extra neural soft tissues and the brain is heavily unidirectional. It is much more common to have a primary somatic malignancy with metastases to the brain than a primary brain tumour with extra neural somatic spread.^1^ In fact, Bailey and Cushing stated in 1926 that primary central nervous system (CNS) tumours never spread outside of the CNS. While this statement has since been debunked, primary CNS tumours have a reported extra neural spread rate of 4.3%.^2^ Conversely, 20–35% of primary somatic tumours have brain metastases.^2^

Haemangiopericytomas (HPCs), also known as solitary fibrous tumours, were first described by Stout and Murray in 1942 as a unique vascular tumour that originated from the Zimmermann pericytes surrounding capillaries and post-capillary venules.^1^ HPCs have been described in all age groups, with peak incidences (more than 40%) occurring in the fifth and sixth decades of life.^3^ The female to male ratio is approximately 2:1.^4,5^ It occurs more commonly in the musculoskeletal system, retroperitoneum and skin, but rarely in the CNS, accounting for only 0.4% of primary CNS tumours and 2% of meningeal tumours.^2,4^ Herein, we report a case of a patient with recurrent primary CNS HPC with extensive systemic metastases.

## Clinical presentation

A 55-year-old ethnic Malay female presented to the emergency department with severe abdominal pain and weight loss. She was conscious and alert. Physical examination on admission revealed an enlarged liver and left kidney as well as a palpable midline abdominal mass. No focal neurological deficit was detected on examination. Her medical history was remarkable for a primary CNS HPC (WHO Grade III) which had been resected 19 years ago. She underwent laminectomy and excisional biopsy of a pathologically proven bony sacral metastases from HPC 8 years prior, with adjuvant radiotherapy to the spine (total dose of 50 Gy given in 25 fractions of 2 Gy each). Further, she had undergone multiple cranial resections for local CNS tumour recurrence (10 and 3 years prior), yet never received radiotherapy to the brain.

### Prior investigations/Imaging findings

**Figure 1.**
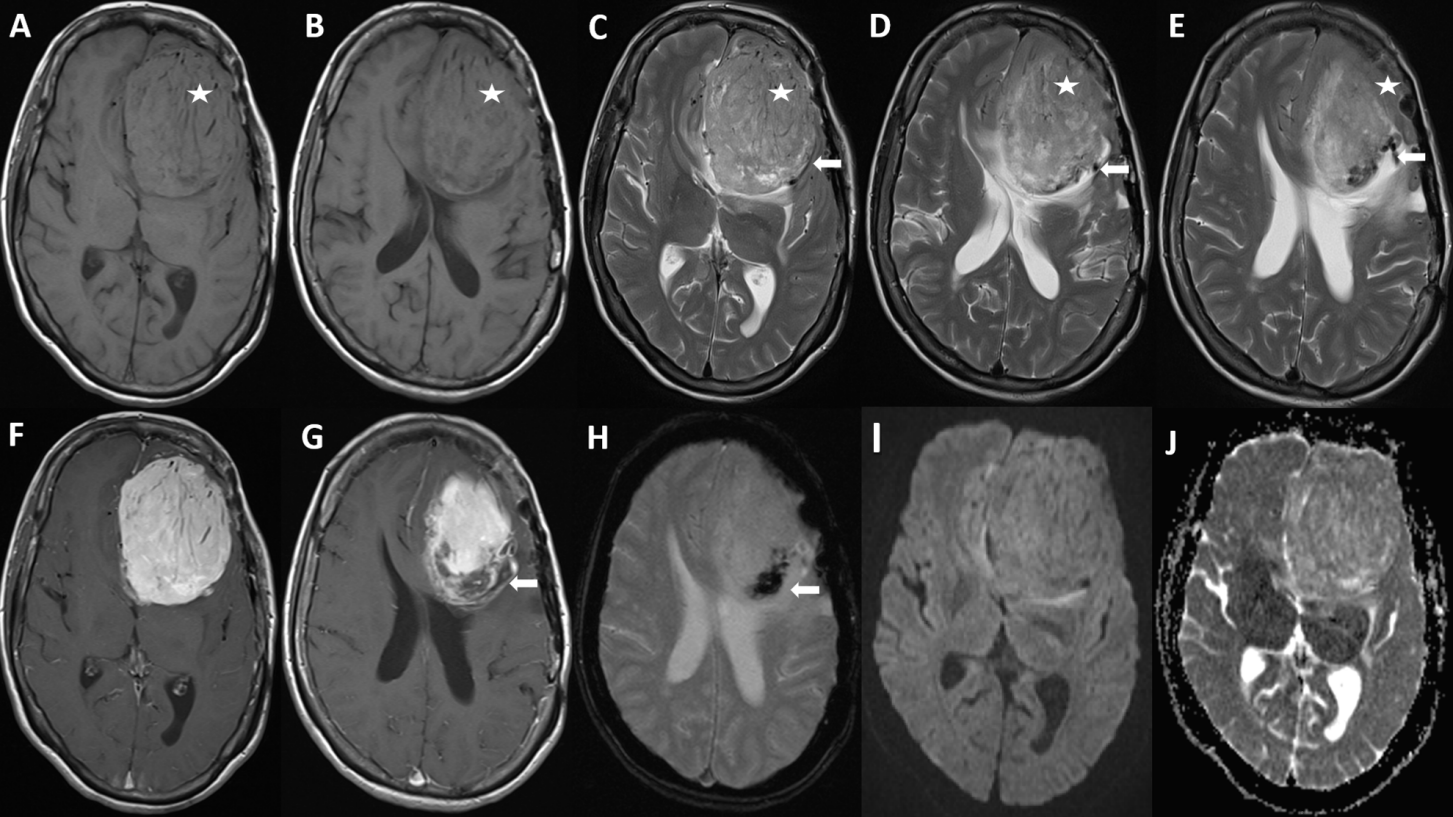
Axial *T*_1_W (A, B) and *T*_2_W (C, D, E) MR images show a large *T*_1_W isointense and heterogeneous *T*_2_W extra axial mass with low *T*_2_W signal areas (stars). The lesion shows avid enhancement following contrast administration (F, G), and a narrow zone of attachment with the dura, mushrooming centrally towards the brain parenchyma. The lesion contains multiple internal flow voids on axial *T*_2_W (C, D, E), post-contrast (G) and GRE (H) images which is consistent with a highly vascularized tumour (arrows). There is no signal change on DWI (I) and ADC (J) sequences to indicate restricted diffusion within the lesion. The constellation of MRI features is compatible with a (recurrent) intracranial haemangiopericytoma. ADC, apparent diffusion coefficient; DWI, diffusion weighted imaging; GRE, gradient echo.

**Figure 2.**
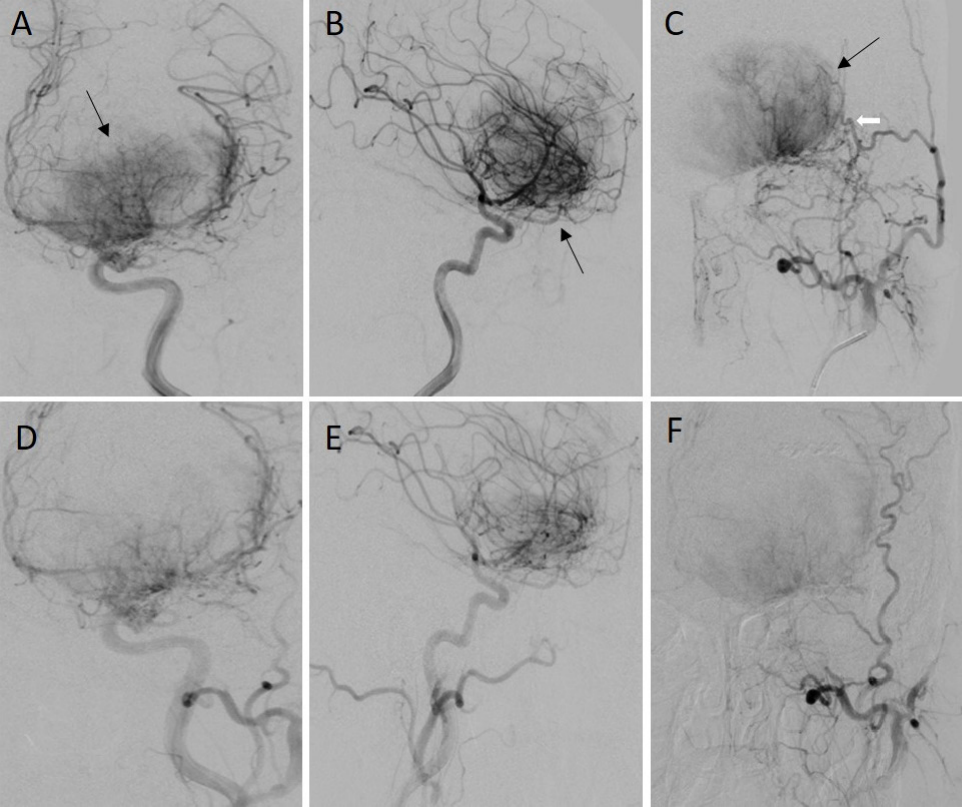
IADSA of the left ICA injections show a significant tumour blush of the left frontal mass in the frontal (A) and lateral (B) projections (black arrows). The mass derives its main arterial supply from the anterior falcine and meningeal branches of the left ophthalmic and left anterior temporal arteries. Left ECA injection (C) shows additional arterial supply to the mass from the zygomatico-orbital branches (white arrow) of the left superficial temporal artery. Pre-operative embolization was subsequently performed in the same sitting using PVA particles with significant reduction of the tumour blush on repeat angiogram following the left ICA (D, E) and ECA (F) injections. ECA, external carotid artery; IADSA, intra-arterial digital subtractioncerebral angiography; ICA, internal carotid artery; PVA, polyvinyl alcohol.

MRI brain 3 years ago revealed a large heterogeneously enhancing extra axial mass in the left frontal convexity at the site of the original operative bed (Figure 1). Pre-operative intra-arterial cerebral angiogram and subsequent polyvinyl alcohol embolization resulted in marked reduction tumour vascularity (Figure 2). She underwent re-craniotomy with gross total resection of the tumour. Histopathological specimens of the resected tumour revealed recurrent HPC, of similar grade to the original tumour (WHO Grade III). Immunohistochemistry was positive for CD99 and BCL2 and negative for S100, EMA and MNF116; no genomics panel was performed.

**Figure 3.**
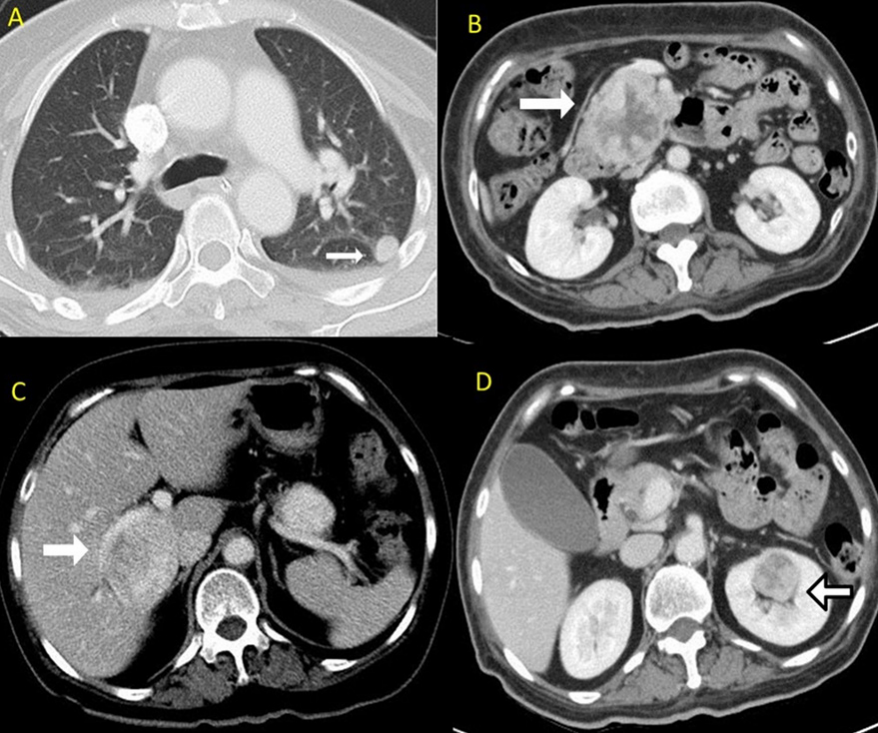
Axial CT of the thorax in the lung window (A) shows a nodular lesion in the apical segment of the left lower lobe (arrow). Axial contrast-enhanced CT scans of the abdomen in the portal venous phase (B, C, D) demonstrate multiple heterogeneously enhancing lesions in the uncinate process of the pancreas (B, arrow), segment VI/VII of the liver (C, arrow) and left kidney (D, arrow). No arterial phase imaging was performed. All the aforementioned lesions in the lung and abdomen indicate metastases from a primary CNS haemangiopericytoma. CNS, central nervous system.

Post-operative MRI of the brain approximately 2.7 years prior to current admission showed no evidence of recurrent intracranial tumour. Concurrent surveillance whole body CT imaging revealed a soft tissue nodule in the apical segment of the left lower lobe and several enhancing lesions in the liver, left kidney, pancreatic head and uncinate process (Figure 3). All the lesions demonstrated similar imaging characteristics to that of the intracranial HPC, and as such were deemed likely to represent extracranial metastases. Hypervascular metastases from a second primary malignancy were considered and histological correlation was advised. The patient, however, refused surgical exploration or biopsy of the pulmonary and abdominal lesions as she was asymptomatic. She was then lost to follow-up until her current admission.

### Investigations and imaging findings of current admission

**Figure 4.**
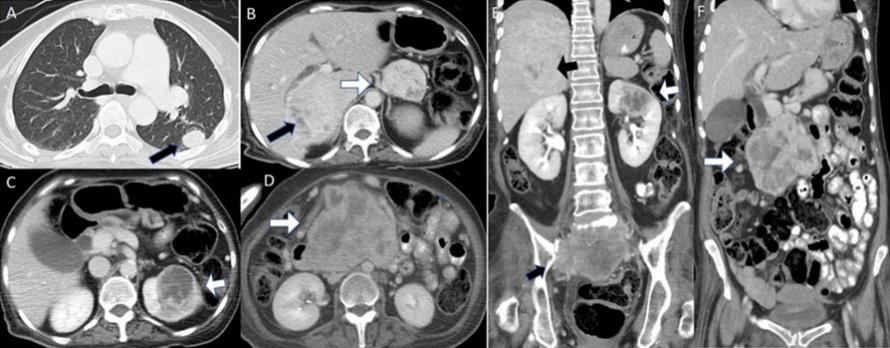
Follow-up CT thorax in the lung window (3 years later after defaulting treatment) shows enlargement of the nodular lesion in the apical segment left lower lobe (A, arrow). Follow-up axial and coronal contrast-enhanced CT scans of the abdomen in the portal venous phase show enlargement of heterogeneously enhancing lesions in hepatic segment VI/VII (B, E, black and white arrows), left kidney (C, E, white arrows) and pancreas head and uncinate process (B, D, F, white arrows). Also, note is made of the recurrent tumour in the sacrum (E, black arrow). These findings are consistent with tumour progression from metastatic spread of haemangiopericytoma.

**Figure 5.**
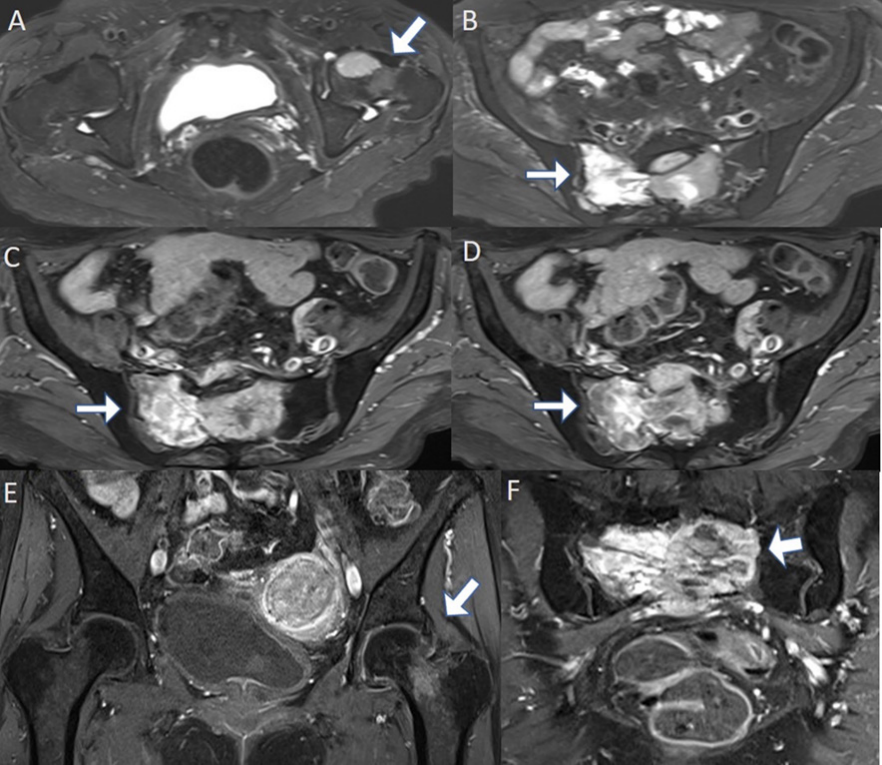
Axial *T*_2_W fat saturated (A, B) and axial (C, D) and coronal (E, F) *T*_1_W post-contrast (fat saturated) MR images of the pelvis show *T*_2_ hyperintense and enhancing tumour involving the left femoral neck and sacrum (arrows). These are consistent with osseous metastases of primary CNS haemangiopericytoma. CNS, central nervous system

Present day laboratory findings revealed severe anaemia, abnormal liver function tests and elevated serum creatinine levels. There were elevated serum lipase and CA 19.9 levels indicating an abnormality of the pancreas. No hypoglycaemia was observed to indicate a paraneoplastic effect from the tumour. CT of the abdomen (Figure 4) revealed interim enlargement of the known lesions in the liver, left kidney and pancreas, consistent with disease progression. The partially imaged vertebrae showed recurrent bony metastases in the sacrum. MRI pelvis (Figure 5) subsequently confirmed recurrent and new bony metastases in the sacrum and left femoral neck, respectively.

### Outcome and follow-up

The patient died in the hospital within 1 week of the last admission. Histopathological examination of the autopsy specimens confirmed the presence of metastatic HPCs in the liver, left kidney, pancreas and bones. No recurrent intracranial tumour was detected.

### Differential diagnoses

Extra axial dural-based tumours which may show similar characteristics to intracranial HPC include meningioma, plasmacytoma and highly vascular dural metastases (such as from malignant melanoma, renal, and breast carcinoma).

Brain tumours which are associated with extracranial spread include medulloblastomas, ependymomas, glioblastoma multiforme (GBM) and gliosarcomas; these tumors are often intra axial in location. Medulloblastomas and ependymomas typically occur in children, while GBM and HPCs are seen in adults.

The differential diagnosis for multiorgan hypervascular abdominal masses would include metastases from primary sources such as renal, lung, melanoma, colon, breast and neuroendocrine tumours. For the latter, the use of specific blood or urine biomarkers (*e.g.* chromagranin A, serotonin, 5-HIAA, glucagon, somatostatin, IGF-I), may help to differentiate NET from metastatic HPC in uncertain cases short of a histologic confirmation.^6^

### Imaging characteristics of intracranial HPC and features that distinguish them from meningiomas

Intracranial HPCs are often misdiagnosed as meningiomas, as they are both extra axial tumours which are attached to the dura and avidly enhance after contrast administration. Careful radiologic inspection of the tumour may help to prospectively distinguish the two entities. Compared to meningiomas, HPCs tend to be more multilobulated and possess a narrow dural attachment and mushroom centrally towards the brain parenchyma, whereas meningiomas tend to have a smoother contour with a wider dural attachment.^7^

On CT, HPCs rarely demonstrate calcification and tend to erode the adjacent bone whereas meningiomas often calcify and may cause bony hyperostosis.^7,8^ On MRI, the *T*_1_W and *T*_2_W signal intensity of HPCs and meningiomas may be quite heterogeneous based on the composition of the tumour and degree of anaplasia, and therefore these tumours not easily distinguished utilising differences in signal intensity. However, HPCs tend to be more vascular than meningiomas, with larger intratumoral flow voids, best identified on *T*_2_W and susceptibility weighted images.^7^ Two-thirds of HPCs will show regions of restricted diffusion on DWI.^8^

To the best of the authors’ knowledge, there are no studies dedicated specifically towards the imaging appearance of recurrent HPC. However, from a few selected images from papers investigating the general imaging appearance of HPCs, the imaging features of recurrent HPC seem be similar to *de novo* HPC (intense contrast enhancement, a narrow zone of attachment to the dura, lobular margins, and marked tumour vascularity).^9^ Our experience with the current case also corroborates recurrent HPC having similar imaging characteristics to its *de novo* counterpart.

### Immunohistochemistry

Immunohistochemistry may be helpful to distinguish between HPC and meningioma. Solitary fibrous tumours are typically positive for STAT 6, bcl-2, CD 34, CD 99 and negative for EMA and S100. Meningiomas, on other hand, tend to be positive for EMA and S100 and negative for the other aforementioned markers.^10^ Note should be made that immunohistochemistry markers vary amongst different subgroups of HPCs, and no marker is 100% sensitive and specific.^11^

## Discussion

Extra neural metastases of intracranial tumours are rare for a number of reasons. The physical hurdles inherent to the cerebral environment, such as the blood–brain barrier and dense dural membrane, makes penetration by tumour cells difficult.^12,13^ Further, a non-hostile CNS environment does little to promote the selection of subpopulations of cells capable of metastases.^2^ Finally, highly aggressive primary CNS tumours such as GBM shorten an expected life span to months or a few years, limiting the potential time these tumours have to spread elsewhere.^2^

HPCs are unique CNS tumours characterised by high rates of local recurrence and distant systemic metastases, as frequently as 70 and 27%, respectively.^4^ HPC metastases typically occur 5–10 years after diagnosis, however, metastasis can be delayed by 20 years after initial diagnosis.^14,15^ Likewise, in our case, there were distant metastases of HPC detected in the sacrum and abdominal organs (liver, pancreas and kidneys) 11 and 16 years, respectively, after the initial diagnosis of meningeal HPC. These extracranial metastatic sites are concordant with those described in the literature in which the bone and the liver are the most common sites of metastases detected in 82 and 41% of cases respectively.^16^ Other common locations for distant HPC metastasis are the lungs (in 29% of cases) and kidneys.^16,17^ Involvement of breast, thyroid and pancreas have rarely been reported.^17,18^

The extensive systemic metastases of HPC in our patient were presumably related to breaching of the blood–brain barrier following multiple cranial surgeries when direct tumour access to the extracranial tissue was made possible via disruption of the dura matter and vessels with subsequent haematogenous systemic spread.^18,19^ Various other mechanisms have been hypothesized for extracranial metastases of intracranial tumours. Radiation may cause direct damage to the natural barriers of the brain and also lead to sarcomatous metaplasia of tumour cells, making them more prone to metastasize. Tumour neovascularization and subsequent haematogenous spread has likewise been implicated.^12^ Lastly, systemic dissemination of tumour cells by the lymphatic system may constitute a possible route for tumour spread given the accumulating evidence pointing to the existence of intradural lymphatic vessels in the brain.^20^

### Treatment and prognosis

Prognosis is significantly dependent on the WHO grade of the HPC. Treated WHO Grade II tumours confer an overall mean survival of 21.3 years and a recurrence free interval of 7.9 years, while treated anaplastic WHO Grade III tumours result in an overall mean survival of 9.5 years and recurrence free interval of 4.9 years.^21^ Although WHO Grade II HPCs outnumber Grade III tumours by 9:1, both of them are able to metastasize out of the CNS; Grade III tumours are, however, 2.5 times more likely to metastasize than their lower grade counterpart.^9,22,23^

Complete surgical excision of tumour is the mainstay of treatment, as several studies have demonstrated an overall survival benefit of gross total resection over subtotal resection. Therefore, more aggressive means to achieve complete surgical excision may be warranted.^21,24^ Embolization, as shown in our case, may be useful before resection to decrease tumour vascularity and associated blood loss encountered by the surgeon. Adjuvant post-operative radiotherapy is also beneficial as it prolongs the recurrence free interval, which may greatly affect the patient’s quality of life.^21^ However, postoperative adjuvant radiotherapy has not shown to definitely prolong overall survival.^21,24^

### Monitoring for extracranial metastases

Heightened suspicion for metastases should be given to all patients with a history of intracranial HPC, regardless of treatment history; the risk of extracranial metastases is not decreased by the extent of intracranial tumour resection or addition of adjuvant radiation. Patient demographics and intracranial tumor location also have no statistical bearing on the development of extracranial HPC metastases.^23^

Unfortunately, there is lack of clear consensus and quality evidence on the frequency and type of imaging surveillance patients with intracranial HPC should undergo to evaluate for systemic metastases. Some authors have recommended annual chest, abdomen, and pelvis CT or whole body positron emmision tomography (PET) CT to monitor for extracranial metastases. However, yearly surveillance may be excessive and not recommended for young patients, given the accumulated radiation dose would increase the risk of future malignancies. Other authors have advocated lower radiation approaches to selected body regions (6–12 month interval chest X rays to evaluate for lung metastases).^23^ Ratneswaren et al performed a meta-analysis of 904 HPC cases documented over 71 studies, and ultimately recommended periodic, life-long clinical surveillance with need for early imaging in patients with symptoms of recurrent disease/metastasis; the choice of imaging modality (X-ray, CT, or MRI) would be dependent on the body region of clinical concern. This recommendation is based on the knowledge that most reported HPC metastases were initially clinically symptomatic before subsequent imaging was performed to confirm metastases.^23^

Aside from fludeoxyglucose PET-CT, other molecular imaging techniques have shown promise towards detecting extracranial HPC metastases because of their ability to efficiently image the whole body.^25,26^ There is some evidence that not all HPC metastases will be reliably hypermetabolic on fludeoxyglucose PET-CT, but instead may demonstrate avidity for somatostatin analogs, such as ^68^Ga DOTATATE and ^111^In-penetreotide.^25,26^ Therefore, somatostatin receptor imaging is yet another adjunctive tool in the clinician’s armamentarium.

## Conclusion

While it is common for primary somatic malignancies to metastasize to the brain, it is relatively rare for primary brain tumours to spread outside of the CNS. HPCs are one of the few primary CNS malignancies that are able to disseminate throughout the body, which may occur years after initial diagnosis. The extensive systemic metastases of meningeal HPC seen in our patient may have been related to breaching of the blood brain barrier following multiple cranial surgeries with subsequent haematogenous systemic spread. Regardless of aetiology, comprehensive measures must be taken to prevent locoregional relapse and the detection of systemic metastases.

## Learning points

1. Intracranial HPC is a rare tumour characterized by the propensity to recur locally and metastasize outside the CNS, which may occur years or even decades after initial tumour diagnosis.
2. Aggressive treatment is imperative for local tumour control, inclusive of gross total resection and adjuvant radiotherapy; long-term post-treatment surveillance is paramount to detect locoregional relapse and systemic metastases.

## References

[1] Stout AP, Murray MR Hemangiopericytoma: a vascular tumor featuring Zimmermann's pericytes. Ann Surg 1942; 116: 26–33. doi: 10.1097/00000658-194207000-00004 17858068 PMC1543753

[2] Subramanian A, Harris A, Piggott K, Shieff C, Bradford R Metastasis to and from the central nervous system--the 'relatively protected site'. Lancet Oncol 2002; 3: 498–507. doi: 10.1016/S1470-2045(02)00819-7 12147436

[3] Kalokhe G, Grimm SA, Chandler JP, Helenowski I, Rademaker A, Raizer JJ Metastatic glioblastoma: case presentations and a review of the literature. J Neurooncol 2012; 107: 21–7. doi: 10.1007/s11060-011-0731-1 21964740

[4] Ravenel JG, Goodman PC Late pulmonary metastases from hemangiopericytoma of the mandible: unusual findings on CT and MR imaging. AJR Am J Roentgenol 2001; 177: 244–6. doi: 10.2214/ajr.177.1.1770244 11418437

[5] Kusumoto S, Nakamura R, Mizoguchi N, Ono S, Watanabe K Primary intrathoracic extrapulmonary hemangiopericytoma. CT and Mr findings. Clin Imaging 1997; 21: 51–3. doi: 10.1016/0899-7071(95)00061-5 9117932

[6] Aluri V, Dillon JS Biochemical testing in neuroendocrine tumors. Endocrinol Metab Clin North Am 2017; 46: 669–77. doi: 10.1016/j.ecl.2017.04.004 28760232 PMC5777173

[7] Ma C, Xu F, Xiao Y-D, Paudel R, Sun Y, Xiao E-H Magnetic resonance imaging of intracranial hemangiopericytoma and correlation with pathological findings. Oncol Lett 2014; 8: 2140–4. doi: 10.3892/ol.2014.2503 25289095 PMC4186565

[8] Lyndon D, Lansley JA, Evanson J, Krishnan AS Dural masses: meningiomas and their mimics. Insights Imaging 2019; 10: 11. doi: 10.1186/s13244-019-0697-7 30725238 PMC6365311

[9] Pang H, Yao Z, Ren Y, Liu G, Zhang J, Feng X Morphologic patterns and imaging features of intracranial hemangiopericytomas: a retrospective analysis. Onco Targets Ther 2015; 8: 2169-78. doi: 10.2147/OTT.S85971 26347312 PMC4550184

[10] Geramizadeh B, Marzban M, Churg A Role of immunohistochemistry in the diagnosis of solitary fibrous tumor, a review. Iran J Pathol 2016; 11: 195–203. 27799967 PMC5079451

[11] Manatakis DK, Delis SG, Ptohis N, Korkolopoulou P, Dervenis C Multidisciplinary approach to hepatic metastases of intracranial hemangiopericytoma: a case report and review of the literature. Case Rep Oncol Med 2015; 2015: 1–5. doi: 10.1155/2015/214306 PMC4450339 26090247

[12] Rosen J, Blau T, Grau SJ, Barbe MT, Fink GR, Galldiks N Extracranial metastases of a cerebral glioblastoma: a case report and review of the literature. Case Rep Oncol 2018; 11: 591–600. doi: 10.1159/000492111 30283316 PMC6167720

[13] Robert M, Wastie M Glioblastoma multiforme: a rare manifestation of extensive liver and bone metastases. Biomed Imaging Interv J 2008; 4: e3. doi: 10.2349/biij.4.1.e3 21614314 PMC3097703

[14] Ding D, Sheehan JP Intracranial hemangiopericytomas: a wolf in sheep's clothing. World Neurosurg 2014; 82(1-2): e185–6. doi: 10.1016/j.wneu.2014.02.019 24549023

[15] Spatola C, Privitera G Recurrent intracranial hemangiopericytoma with extracranial and unusual multiple metastases: case report and review of the literature. Tumori 2004; 90: 265–8. doi: 10.1177/030089160409000222 15237597

[16] Galanis E, Buckner JC, Scheithauer BW, Kimmel DW, Schomberg PJ, Piepgras DG Management of recurrent meningeal hemangiopericytoma. Cancer 1998; 82: 1915–20. doi: 10.1002/(SICI)1097-0142(19980515)82:10<1915::AID-CNCR15>3.0.CO;2-W 9587125

[17] Kim JH, Jung H-W, Kim Y-S, Kim CJ, Hwang S-K, Paek SH, et al Meningeal hemangiopericytomas: long-term outcome and biological behavior. Surg Neurol 2003; 59: 47–53. doi: 10.1016/s0090-3019(02)00917-5 12633961

[18] Hiraide T, Sakaguchi T, Shibasaki Y, Morita Y, Suzuki A, Inaba K, et al Pancreatic metastases of cerebellar hemangiopericytoma occurring 24 years after initial presentation: report of a case. Surg Today 2014; 44: 558–63. doi: 10.1007/s00595-012-0415-2 23180115

[19] Siegel HJ, Lopez-Ben R, Sutton JH, Siegal GP Intracranial meningeal hemangiopericytoma metastatic to the scapula. Orthopedics 2012; 35: e112–5. doi: 10.3928/01477447-20111122-37 22229602

[20] Sun B-L, Wang L-H, Yang T, Sun J-Y, Mao L-L, Yang M-F, et al Lymphatic drainage system of the brain: a novel target for intervention of neurological diseases. Prog Neurobiol 2018; 163-164: 118–43. doi: 10.1016/j.pneurobio.2017.08.007 28903061

[21] Melone AG, D'Elia A, Santoro F, Salvati M, Delfini R, Cantore G, et al Intracranial hemangiopericytoma--our experience in 30 years: a series of 43 cases and review of the literature. World Neurosurg 2014; 81(3-4): 556–62. doi: 10.1016/j.wneu.2013.11.009 24239740

[22] Zweckberger K, Jung CS, Mueller W, Unterberg AW, Schick U Hemangiopericytomas grade II are not benign tumors. Acta Neurochir 2011; 153: 385–94. doi: 10.1007/s00701-010-0877-1 21104099

[23] Ratneswaren T, Hogg FRA, Gallagher MJ, Ashkan K Surveillance for metastatic hemangiopericytoma-solitary fibrous tumors-systematic literature review on incidence, predictors and diagnosis of extra-cranial disease. J Neurooncol 2018; 138: 447–67. doi: 10.1007/s11060-018-2836-2 29551003

[24] Rutkowski MJ, Sughrue ME, Kane AJ, Aranda D, Mills SA, Barani IJ, et al Predictors of mortality following treatment of intracranial hemangiopericytoma. J Neurosurg 2010; 113: 333–9. doi: 10.3171/2010.3.JNS091882 20367074

[25] Kota G, Gupta P, Lesser GJ, Wilson JA, Mintz A Somatostatin receptor molecular imaging for metastatic intracranial hemangiopericytoma. Clin Nucl Med 2013; 38: 1–987. doi: 10.1097/RLU.0000000000000237 24212443

[26] Hung T-J, Macdonald W, Muir T, Celliers L, Al-Ogaili Z 68Ga DOTATATE PET/CT of Non-FDG-Avid pulmonary metastatic hemangiopericytoma. Clin Nucl Med 2016; 41: 779–80. doi: 10.1097/RLU.0000000000001313 27454598

